# Relevance of Polymorphic KIR and HLA Class I Genes in NK-Cell-Based Immunotherapies for Adult Leukemic Patients

**DOI:** 10.3390/cancers13153767

**Published:** 2021-07-27

**Authors:** Léa Dubreuil, Patrice Chevallier, Christelle Retière, Katia Gagne

**Affiliations:** 1 Department of Research, Etablissement Français du Sang, F-44011 Nantes, France; lea.dubreuil@efs.sante.fr (L.D.); christelle.retiere@efs.sante.fr (C.R.); 2 University de Nantes, INSERM U1232 CNRS, CRCINA, F-44000 Nantes, France; patrice.chevallier@chu-nantes.fr; 3 LabEx IGO “Immunotherapy, Graft, Oncology”, F-44000 Nantes, France; 4 Hematology Clinic, CHU, F-44093 Nantes, France; 5 LabEx Transplantex, Université de Strasbourg, F-67000 Strasbourg, France

**Keywords:** natural killer cells, killer-cell immunoglobulin-like receptors, HLA class I, polymorphism, acute leukemia, hematopoietic stem-cell transplantations, NK-cell-based immunotherapy

## Abstract

**Simple Summary:**

Immunotherapies are promising approaches to curing different acute leukemias. Natural killer (NK) cells are lymphocytes that are efficient in the elimination of leukemic cells. NK-cell-based immunotherapies are particularly attractive, but the landscape of the heterogeneity of NK cells must be deciphered. This review provides an overview of the polymorphic KIR and HLA class I genes that modulate the NK cell repertoire and how these markers can improve the outcomes of patients with acute leukemia. A better knowledge of these genetic markers that are linked to NK cell subsets that are efficient against hematological diseases will optimize hematopoietic stem-cell donor selection and NK immunotherapy design.

**Abstract:**

Since the mid-1990s, the biology and functions of natural killer (NK) cells have been deeply investigated in healthy individuals and in people with diseases. These effector cells play a particularly crucial role after allogeneic hematopoietic stem-cell transplantation (HSCT) through their graft-versus-leukemia (GvL) effect, which is mainly mediated through polymorphic killer-cell immunoglobulin-like receptors (KIRs) and their cognates, HLA class I ligands. In this review, we present how KIRs and HLA class I ligands modulate the structural formation and the functional education of NK cells. In particular, we decipher the current knowledge about the extent of KIR and HLA class I gene polymorphisms, as well as their expression, interaction, and functional impact on the KIR^+^ NK cell repertoire in a physiological context and in a leukemic context. In addition, we present the impact of NK cell alloreactivity on the outcomes of HSCT in adult patients with acute leukemia, as well as a description of genetic models of KIRs and NK cell reconstitution, with a focus on emergent T-cell-repleted haplo-identical HSCT using cyclosphosphamide post-grafting (haplo-PTCy). Then, we document how the immunogenetics of KIR/HLA and the immunobiology of NK cells could improve the relapse incidence after haplo-PTCy. Ultimately, we review the emerging NK-cell-based immunotherapies for leukemic patients in addition to HSCT.

## 1. Introduction

Natural killer (NK) cells, which develop from common lymphoid progenitors in the bone marrow, correspond to large granular lymphocytes and are usually identified as CD3^−^ CD56^+^, representing about 5–20% of peripheral blood mononuclear cells (PBMCs) [1]. These innate immune effectors [2] proliferate, produce cytokines, and are cytotoxic against virus-infected cells or tumor cells. According to the level of CD56 expression, two NK cell subsets with distinct repertoire signatures have been defined; CD56^dim^ NK cells (90%) are mainly cytotoxic, while CD56^bright^ NK cells (10%) produce high levels of cytokines, but have a low cytotoxicity [3]. NK cells are able to lyse target cells by using antibody-dependent cellular cytotoxicity (ADCC) driven by the activating CD16 receptor. They express a wide range of activating receptors including NKG2D, DNAM-1, 2B4, and NCRs such as NKp30, NKp44, and NKp46 that recognize induced or upregulated molecules on tumor and virus-infected cells. NK cells recognize healthy cells that express normal levels of HLA class I molecules and sense the absence of HLA class I molecules on allogeneic cells or their decreased expression on tumor or virus-infected cells, as demonstrated by the “missing-self” hypothesis [4,5]. Killer-cell immunoglobulin-like receptors (KIRs) and the CD94/NKG2A heterodimer represent the two main inhibitory NK receptors that are engaged in this missing-self recognition. Overall, NK cell functions are regulated by a balance of synergistic signals from combinations of inhibitory and activating receptors engaged with cognate ligands on target cells. HLA class I molecules play a central role in the regulation of immune responses with respect to their interactions with T- or NK-cell-specific receptors [6]. KIRs and their HLA class I ligands constitute the main polymorphic and polygenic pair in humans for the regulation of functions and the licensing of NK cells [6,7]. KIRs are clonally expressed on the surface of mature NK cells [8,9]. Functional KIR/HLA interactions drive the tremendous diversity of NK cells with a limited number of germline-encoded genes [10]. In addition to KIRs, the CD94/NKG2A heterodimer recognizes the nonclassical HLA-E molecule. This receptor/ligand is expressed in all individuals and does not present the broad genetic diversity that characterizes the KIR/HLA pair. The inhibitory CD94/NKG2A receptor and its activating NKG2C counterpart are involved in missing-self recognition, and CD94/NKG2A is also involved in NK cell licensing [11]. Moreover, the expression of inhibitory and activating receptors is strongly associated with NK cell differentiation and maturation. In particular, NK cell subsets can be defined on the basis of differentiation markers. During their development, NK cells first acquire NKG2A expression and, at a later stage, KIR expression, followed by the terminal differentiation CD57 receptor [11]. The cytomegalovirus (CMV) also modulates the NK cell repertoire with an expansion of KIR2D^+^ NKG2C^+^ NK cells [12,13]. The model of NK cell differentiation established by Björsktrom et al. [11], therefore, permits the investigation of the heterogeneity of NK cells.

In cancer immunotherapies, NK cells represent key components especially for patients with hematological malignancies. In these cases, NK cells can easily access tumor sites without the barrier represented by the tumor microenvironment, unlike in the case of solid tumors [14]. Although they are diverse, hematological malignancies include acute myeloid leukemia (AML) and acute lymphoid leukemia (ALL), which are frequent in adult patients. For patients with acute leukemia, the relapse of the initial disease remains the major cause of death. The relapse may be due to a failure of conventional chemotherapies but also due to the escape of tumor cells from immune responses [15]. The lost or decreased expression of HLA molecules and/or upregulation of ligands on leukemic cells that are recognized by activating NK cell receptors can sustain the crucial role of NK cells. For leukemic patients for whom conventional chemotherapies have failed, allogeneic hematopoietic stem-cell transplantation (HSCT) is usually suggested in order to replace both the defective hematopoiesis and the immune system of the patient with healthy HSCs from donors. In that case, NK cells play a crucial role through their graft-versus-leukemia (GvL) effect, as they are the first lymphocytes to participate in the reconstitution of the immune system of the patient even before T cells after HSCT [16,17]. However, the NK cell functions in leukemic patients can be altered by the tumor microenvironment. The efficiency of NK-cell-based immunotherapies can be enhanced by cytokines, inhibitory specific monoclonal antibodies (mAbs), and adoptive transfer of in vitro activated NK cells [18,19]. Promising recent advances have shown the use of NK cells armed with chimeric antigen receptors (CARs) to enhance their antileukemic function [20]. The CRISPR technology also permits the optimization of some cellular pathways to boost the antileukemic efficacy of NK cell immunotherapies [21]. Numerous approaches to NK cell immunotherapy have been developed by using mainly autologous or allogeneic NK cells in bulk without considering all genetic and allelic KIR and HLA polymorphisms known to govern the structural and functional heterogeneity of NK cells.

**Figure 1:**
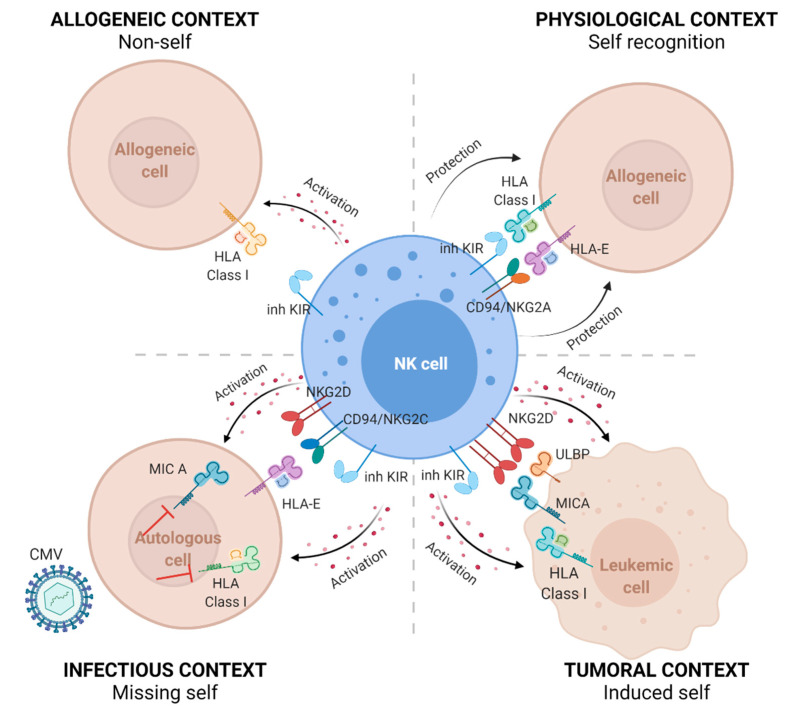
The natural cytotoxicity of NK cells is driven by the missing-self recognition. NK cells recognize healthy cells that express normal levels of HLA class I molecules and sense the absence of HLA class I molecules on allogeneic cells (non-self) or decreased expression of HLA class I molecules (missing-self) on tumor cells or virus-infected cells, as reported for the cytomegalovirus (CMV). Recognition of “self” mainly involves interactions between polymorphic inhibitory killer-cell immunoglobulin-like receptors (inh KIRs) and classical HLA class I molecules, as well as interactions between the CD94/NKG2A heterodimer and the nonclassical HLA-E molecule. The lack of interaction of inh KIRs with their HLA class I ligands or CD94-NKG2A with HLA-E induces the activation of NK cell lysis. In addition to KIR/KIR ligands, interactions of the induced MHC class I-like MICA ligand, ULBP ligand expressed on tumor cells, or the HLA-E molecule by respectively activating NKG2D or CD94/NKG2C receptors could activate NK cell lysis.

In the present review, we present the characteristics of KIR and HLA class I gene polymorphisms, their expression, the KIR/HLA interactions that regulate the NK cell repertoire in healthy individuals, and the relevance of KIR/KIR ligands to NK-cell-based immunotherapies for adult patients with hematological malignancies, especially in the context of allogeneic HSCT. The roles of NK cells in physiological, allogeneic, infectious, and leukemic contexts are summarized in Figure 1.

## 2. Relevance of the KIR and HLA Class I Genes to the NK Cell Repertoire

In healthy individuals, the formation of the NK cell repertoire is influenced by various factors including the variability of the KIR and HLA class I genes, the CMV status, and the frequency of NK cell subsets. KIRs represent a family of receptors belonging to the immunoglobulin (Ig)-like superfamily. The KIR family is composed of receptors with two or three (2D, 3D) extracellular domains. The intracytoplasmic tail can be long (L) with two ITIMs (immune tyrosine-based inhibitory motifs) or short (S) and coupled with the DAP12 molecule, which bears an ITAM (immune tyrosine-based activating motif) discriminating inhibitory and activating KIRs, respectively [22].

### 2.1. KIR Genes

The genetics of KIRs are complex because KIR genes vary in structure, exon/intron organization, and relative size. Currently, the KIR gene family consists of 15 different loci (2DL1, 2DL2/2DL3, 2DL4, 2DL5A, 2DL5B, 2DS1, 2DS2, 2DS3, 2DS4, 2DS5, 3DL1/3DS1, 3DL2, 3DL3, 2DP1, 3DP1) encoded in a 100–200 kb region in the leucocyte region complex (LRC) located on chromosome 19. The LRC constitutes a dense cluster of immune-related genes that encode, in addition to KIR, other NK cell receptors [23]. KIR genes have a head-to-tail organization and are arranged within 2.4 kb of each other, except between 3DP1 and 2DL4, where there is a gap of 14 kb [24]. KIR genes have four to nine exons with an entire genomic size ranging from 4 kb for 3DP1 to 16 kb for 3DL2 and long introns [25]. Three KIR exon/intron gene organizations have been described. The first one corresponds to the genes encoding KIR3DL, such as 3DL1/2. These genes consist of nine exons: exons 1 and 2 encode the signal peptide; exons 3, 4, and 5 encode each Ig-like domain (D0, D1, and D2 respectively); exon 6 encodes the stem connecting the D2 domain to the transmembrane part, which is encoded by exon 7; exons 8 and 9 encode the intracytoplasmic tail. KIR3DL3 has a similar structure, but with the absence of exon 6 and a stop codon in exon 9. KIR3DS1 has the same exon/intron organization as that of 3DL1, but a shorter exon 9 encoding for the short tail. Interestingly, KIR3DL1 and KIR3DS1 are defined as alleles of the same gene, although they have different functions [26]. The second and third exon/intron gene organization corresponds to KIR2D: type I KIR2D includes genes that encode 2DL1/2/3/S1/2/3/4/5. These genes consist of eight exons with exons 4 and 5 encoding the D1 and D2 domains respectively, and a region homologous to exon 3 encoding the D0 domain of KIR3D, which is called “pseudo-exon 3” [27,28]. The KIR2DS4 gene is found in two forms, with one common 1D variant that is characterized by a 22 bp deletion in exon 5, which block transcription of the corresponding receptor [24]; the type II KIR2D genes include 2DL4/5, which exhibits eight exons and a 22 kb deletion in exon 4. Exons 3 and 5 of these genes encode the D0 and D2 domains, respectively.

The number and nature of KIR genes differ between individuals, allowing one to distinguish two major KIR haplotypes (A, B) [29]. The KIR A and B haplotypes both have four framework genes (3DL3, 3DP1, 2DL4, and 3DL2), and are present in all individuals with some rare exceptions. KIR3DL3 and 3DL2 are located at the centromeric and telomeric ends, respectively, and delimit the LRC, while 3DP1 and 2DL4 are located between both. The KIR A haplotype is defined by a fixed set of nine KIR genes (3DL3, 2DL3, 2DP1, 2DL1, 3DP1, 2DL4, 3DL1, 2DS4, and 3DL2) with only 2DS4 as an activating gene. In contrast, the B haplotypes are diverse with a variable number of KIR genes and are characterized by the presence of specific inhibitory KIRs, such as 2DL2, and more than one activating KIR gene. Based on the combination of the KIR A and B haplotypes, 660 KIR genotypes have been described worldwide (http://www.allelefrequencies.net/) (accessed on 26 July 2021). Conventionally, the gene frequencies of KIR are close to 100% for inhibitory 2DL1/2/3, whereas the frequencies of the activating KIR genes are low, except for 2DS4. Different commercial or in-house methods including the use of specific KIR primers or probes, permit the assessment of the presence or absence of KIR genes. Various studies have shown that frequencies of KIR genes, haplotypes and/or genotypes vary between populations [30,31,32]. Interestingly, a majority (˃50%) of individuals harbors the KIR AA genotype in Asian populations [30,33], whereas only 35% of Europeans have this genotype [34,35].

In addition to the definition of the KIR A and B haplotypes, framework KIR genes allow for the delineation of two chromosomic parts: centromeric (cen) (2DS2, 2DL2/3, 2DL5B, 2DS3, 2DP1, 2DL1) and telomeric (tel) KIR genes (3DL1/S1, 2DL5A, 2DS5, 2DS1) [36]. Depending on the KIR2DL2/3/S2 genes, the cenAA (2DL3 only), cenAB (2DL3 with 2DS2 and/or 2DL2), and cenBB (2DL2 and/or 2DS2, no 2DL3) motifs are defined. Depending on KIR2DS1/S4/3DL1/S1 genes, the telAA (3DL1 and 2DS4 only), telAB (3DL1 and 2DS4 with 3DS1 and/or 2DS1), and telBB (lacking 3DL1 and/or 2DS4) motifs are defined. This subdivision into gene content motifs can increase the diversity of KIR genes. Thus, individuals with a KIR AB genotype could present different cen and tel motifs, whereas individuals with the KIR AA genotype exhibit only the cenAA and telAA motifs. Another parameter of this diversity is linked to the copy number variation (CNV) [37]. The arrangement of KIR genes in a head-to-tail orientation and their high sequence homology allow for a gain or loss of KIR genes with the generation of expanded or contracted KIR haplotypes, as well as the formation of fusion genes [38]. It has been shown that some KIR genes are more affected by CNV than others [33].

Lastly, KIR genes exhibit an allelic diversity; currently, 1110 KIR alleles have been referenced (https://www.ebi.ac.uk/ipd/kir/) (accessed on 26 July 2021). The allelic polymorphism of KIRs is particularly high for inhibitory KIRs (the strongest being for 3DL3 with 228 alleles) and is limited for activating KIRs (the lowest being for 2DS1 with 33 alleles). To name KIR alleles, a specific nomenclature [39] has been established, which uses an asterisk as a separator after the gene name, before a numerical allele designation (2DL1*). The next three digits are used to name the different alleles of the same gene that differ in coding sequences (2DL1*003). The following two digits are used to distinguish alleles that only differ in synonymous (noncoding) substitutions within the coding sequence (2DL1*00302), and the last two digits are used to distinguish alleles that only differ in substitutions in noncoding regions (2DL1*0030202). For more than 15 years, specific KIR primers, probes or sequence-based-typing (SBT) methods have been used to assign KIR alleles of limited KIR genes [40]. More recently, next-generation sequencing (NGS) technologies have permitted the assignment of the alleles of all KIR genes [41,42,43]. As mentioned for KIR genes, KIR allele frequencies vary among populations. Interestingly, although 68 KIR2DL1 alleles have been reported worldwide, only five predominant alleles (*001, *002, *003, *004, and *007) are encountered in Europeans [34,44]. Moreover, the non-expressed KIR3DL1*004 allele [45], which is frequent in Caucasians [46], is absent in the Japanese population [30]. Interestingly, the group of P. Parham was the first to describe that allelic polymorphism of KIRs synergizes with variable gene content, thus making it possible to individualize KIR genotypes [47]. Recently, the KIR A haplotype and cenAA motif were reported to be associated with the 2DL1*001, *002, and *003 alleles, while the B haplotype and cenB+ motif were associated with the 2DL1*004 and *007 alleles in European populations [34,48,49,50]. In addition, different linkage disequilibria (LD) between KIR genes have been described [24,48]. Overall, these population-based genetic studies could help in the prediction of KIR alleles from KIR gene content, including pseudogenes [51].

### 2.2. HLA Class I Genes

The main characteristics of the HLA system correspond to an exhaustive polymorphism and the inheritance of HLA genes from the parental haplotypes in blocks. The HLA system represents the most polymorphic gene locus in humans [52], with 28,938 HLA alleles reported so far [53]. The HLA locus contains more than 200 genes, many of which have immune-related functions [54]. HLA class I genes encode both classical and nonclassical HLA class I molecules. Classical HLA class I genes (HLA-A, -B, -C) are located on the short arm of chromosome 6 (6p21.3) and are composed of eight exons and seven introns. Most polymorphic exons correspond to exons 2 and 3, which respectively, encode the α1 and α2 domains which form the peptide-binding groove of HLA class I molecules that are ubiquitously expressed on nucleated cells. The length of the HLA-A, -B, and -C genes is closed to 3 kb. In terms of allelic polymorphism, HLA-A, -B, and -C are divergent genes with numerous alleles, and all positions in the α1 and α2 domains exhibit variations [55]. The polymorphism of HLA-C genes, although extremely diverse, is more limited in terms of the alleles described in comparison with the polymorphism of HLA-B genes. The allele frequencies of HLA-C and the LD of HLA-C/HLA-B are frequently updated through genetic population studies [56,57,58]. From its phylogeny, HLA-C appears to be more recent compared to the HLA-A and -B loci, and regulatory elements of transcription differ between the HLA-C and HLA-A/B genes [59]. Today, HLA class I alleles are assigned by using NGS methods [57,60,61,62,63], which allow detailed mapping of all polymorphic bases encountered in the exons, introns, and untranslated regions (UTR). Identification of regulatory variants located in UTRs is important because they can influence the expression of HLA class I molecules, as detailed later. In addition to the classical HLA class I genes, the HLA-E and MICA genes, despite their limited polymorphism are of interest in NK cell studies, as they encode for the CD94/NKG2C and NKG2D ligands, respectively [64,65].

### 2.3. KIR/HLA Interactions and the KIR^+^ NK Cell Repertoire

The distribution of KIRs on the surface of NK cells is clonal due to the stochastic expression of KIRs [66,67]. Therefore, a heterogeneous repertoire of NK cell subpopulations expressing different combinations of KIRs or a lack thereof is observed. Usually, the expression of KIRs on NK cells is assessed by using commercial (which is limited to some KIRs) and/or in-house anti-KIR mAbs, which make it possible to distinguish the main inhibitory and activating KIR2D [68]. The KIR gene content influences the KIR^+^ NK cell repertoire. Thus, KIR AA genotyped individuals show a less diverse NK cell repertoire with higher frequencies of the corresponding KIR^+^ NK subpopulations compared to KIR B+ genotyped individuals, who have a broader KIR gene content and lower frequencies of KIR^+^ NK subpopulations [69]. In addition to KIR gene content, the expression of KIR allele encoded products (allotypes) on NK cells may differ depending on the allelic polymorphism, as deeply documented for 3DL1 by P. Parham’s group [70,71]. We also reported high (3DL1*001, *002, *015, *008), low (*005, *007), or no (*004, *019) expression of KIR3DL1 on NK cells depending on the 3DL1 alleles or the 3DL1/S1 allele combinations [46]. The differential expression of KIR3DL1 allotypes could be partially explained by the polymorphisms encountered in the KIR3DL1 promoter [72]. Similarly, three single-nucleotide polymorphisms (SNPs) located in the recruiting sites of transcriptional factors in the KIR2DL1 promoter induce a higher transcriptional activity of 2DL1 compared to 2DL3 [73]. Similarly to KIR3DL1, the expression level of KIR2DL1 is variable depending on the 2DL1 alleles. In particular, the KIR2DL1*004 allotype, which carries a cysteine in the intracytoplasmic tail at position 245 (Cys^245^), is poorly expressed on NK cells, with a low frequency of KIR2DL1^+^ NK cells compared to the 2DL1*002 and *003 (Arg^245^) allotypes [34,74,75].

KIRs interact with HLA class I molecules. Inhibitory KIR2DL1/2/3 all have HLA-Cw molecules as ligands, which are initially divided into two groups as a function of the 77 and 80 amino acids located in the α2 domain. KIR2DL1 specifically recognizes the HLA-Cw molecules of group 2 (C2) with Lys^80^ [76]. The peptide presented by the HLA-Cw molecule seems to play a role in the binding affinity of KIR2DL1/C2 because a substitution at the P8 position with an acidic residue results in a loss of ligand binding [77,78]. KIR2DL2/3 recognize the HLA-Cw molecules of group C1 (C1) with Asp^80^ [79]. However, the recognition of KIR2DL2/3 for C1 is less stringent than the recognition of 2DL1 for C2 because some C2 ligands, such as HLA-Cw4, are also recognized by KIR2DL2/3 [34,80,81]. In addition, the avidity of KIR2DL2 for the C1 ligands is stronger than for 2DL3 [80,82]. Interestingly, the peptide could increase the affinity of KIR2DL2/3 for the C1 ligands, as reported in the context of HCV [83]. KIR2DL4, which is an activating receptor, has the HLA-G molecule as a ligand, which is a nonclassical HLA class I molecule expressed on trophoblast cells during pregnancy [84]. KIR2DS1 recognizes C2 ligands as its inhibitory 2DL1 counterpart, but with a lower affinity [85,86]. KIR2DS2 recognizes C1 ligands and the HLA-A11 molecule [87,88]. As reported for KIR2DL2/3, the recognition of KIR2DS2 of HLA-C molecules is peptide-dependent [88,89]. KIR2DS4 recognizes some HLA-C molecules and the HLA-A11 molecule [90]. To date, the ligands of KIR2DL5 and 2DS3 remain unknown. KIR3DL1 recognizes HLA-B and some HLA-A molecules that bear Bw4 motifs [91,92], while its activating 3DS1 counterpart probably interact with the nonclassical HLA-F molecule [93], although a Bw4 specificity was reported in the context of HIV [94]. KIR3DL2 interacts with HLA-A3/A11 molecules, and this interaction is peptide-dependent, which allows a modulation of receptor ligand interactions [95,96]. In addition to HLA class I ligands, the HERV-H LTR-associating 2 (HHLA2) molecule, a member of the B7 family that is highly expressed in several solid and hematological cancers, was recently identified as a ligand for KIR3DL3 [97]. HLA class I molecules also play a predominant role in NK cell education or licensing. Four models of NK cell education, which allow for the functional development and tolerance to self in NK cells, have been described [98]. Among them, the “arming” or “licensing” model, which was initially described in mice, involves the engagement of an inhibitory NK receptor with a self-MHC class I molecule, and this engagement educates NK cells to become functionally competent. Conversely, the absence of engagement of the inhibitory NK receptor with its ligand induces so-called “unlicensed” NK cells [99]. The education of KIR^+^ NK cells allows these cells to distinguish healthy cells with normal expression levels of HLA class I molecules from those with no or decreased expression of HLA class I molecules, thus referring to the “missing self” theory [7]. Different studies demonstrated the importance of KIR2DL1/2 [7] and 3DL1 [100] in NK cell education. KIR–HLA interactions could also occur in *cis* on NK cells [100]. Overall, NK cell education models are based on inhibitory KIRs, but activating KIRs could also play a role. Indeed, KIR2DS1^+^ NK cells are alloreactive toward C2 targets, but only in C2- individuals [85]. In C2 individuals, KIR2DS1^+^ NK cells become anergic due to the strong and prolonged exposure of 2DS1 to its autologous C2 ligand, which limits the autoreactive immune response [101].

In addition to the phenotype, allelic polymorphisms in KIRs influence the functionality of KIR^+^ NK cells. Thus, the lowest recognition of C2 ligands by KIR2DL1^+^ NK cells was observed for the KIR2DL1*004 allotype [34,74,75]. This decreased recognition was reported to be due to the mutation of Arg^245^ to Cys^245^. Indeed, this mutation plays a role in the transduction of inhibitory signals, as KIR2DL1 allotypes carrying Arg^245^ recruit SHP-2 and β-arrestin 2 molecules, both of which are important signaling molecules mediated by 2DL1 [74]. Recently, we reported that the KIR2DL1*002 allotype (Arg^245^) interacts with all C2 targets as observed with the 2DL1*004 allotype (Cys^245^), suggesting that polymorphic positions other than that of the intracytoplasmic 245 residue may play a crucial role [34]. To a lesser extent, the KIR2DL2/3 allotypes impact the inhibition of KIR2DL2/3^+^ NK cell degranulation, showing a better inhibition with the 2DL2 allotype than with the 2DL3 allotype [102]. The allelic polymorphism of HLA-C could also affect the binding affinity of KIR2DL/HLA-C and the consequent NK cell functionality. Similarly to KIR2D, allelic polymorphisms in KIRs modulate the functionality of KIR3D^+^ NK cells, as was found through extensive studies of KIR3DL1 [30,103,104].

HLA-Cw molecules are more specifically dedicated to sensing the level of alteration and/or decreases in HLA class I expression at the surface of tumor cells than to the presentation of peptides to TcR [105]. As described previously, all HLA-Cw molecules are ligands for KIRs in an allele-specific manner [48,106]. In healthy individuals, the expression level of HLA-Cw molecules on fresh PBMCs is lower than that of HLA-A and -B molecules [105]. The differential expression of HLA-Cw molecules according to tissue specificity must be further investigated. The level of HLA-Cw expression varies depending on the HLA-C alleles, HLA haplotypes and polymorphisms encountered in the coding and regulatory regions of the HLA-C gene [107,108,109,110,111,112]. Genome-wide association studies (GWAS) performed in US blood donors showed a biallelic polymorphism of some SNPs located in the 5′UTR region −800 bp [109] and −35 kb [107] upstream of the initiation codon of the HLA-C gene or in the 3′UTR region [110] that had an effect on the HLA-C expression level. Usually, the expression level of HLA-Cw4 or -Cw6 is higher than that of HLA-Cw2 or -Cw8, but a low expression level was reported for the HLA-Cw7 and -Cw3 molecules. While heterogeneity in the HLA-Cw expression level is observed according to the polymorphisms in the coding and regulatory regions, some discordances remain depending on the population studied (mostly US populations), the methodologies used (e.g., SNPs, evaluation of the HLA-Cw expression level using the HLA-C/HLA-E-specific DT9 mAb, or quantification of mRNA), and the allelic specificities of HLA-C under study. At present, it is difficult to correlate all HLA-C specificities, SNP polymorphisms, levels of HLA-C transcripts, and the corresponding HLA-Cw expression levels in both healthy individuals and people with diseases.

**Figure 2:**
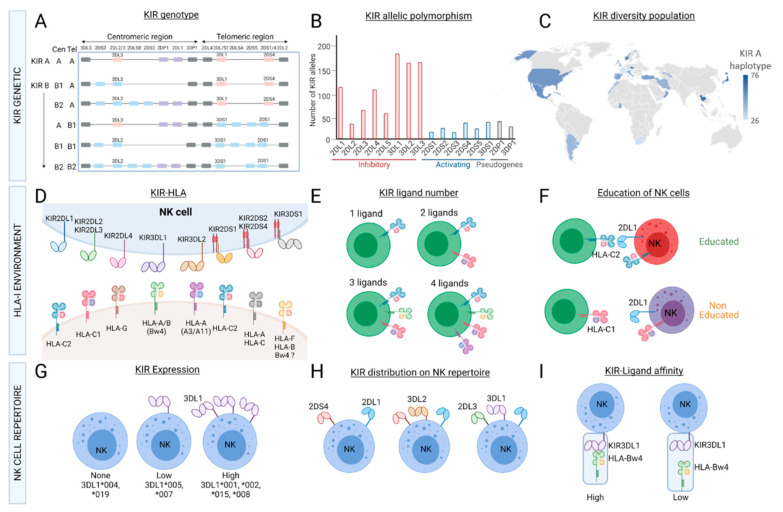
Variability of the KIR^+^ NK cell repertoire. KIR genes (7–14) are located in the leucocyte region complex (LRC) on chromosome 19. Depending on the KIR gene content, different KIR haplotypes (A and B) and centromeric/telomeric motifs are defined (**A**). KIR genes exhibit an allelic polymorphism (https://www.ebi.ac.uk/ipd/kir/) (accessed date: 26 July 2021). (**B**), and the diversity of KIR haplotypes and/or alleles can be observed worldwide (source provided by Dr J. Hollenbach from the 15th IHWG KIR component) (**C**). HLA class I molecules are ligands of KIRs (**D**). Depending on the typing of HLA-A, -B and -C, individuals can present from one to four KIR ligands (**E**). The HLA class I environment also play a major role in NK cell licensing (**F**). The allelic polymorphism of KIRs has an impact on the NK cell repertoire, as illustrated for the expression of different KIR3DL1 allotypes (**G**). KIRs are clonally distributed on NK cells; thus, the heterogeneous repertoire of NK cell subsets is based on the expression of different combinations of KIRs with different frequencies (**H**). The functions (cytotoxicity, cytokine production) of KIR^+^ NK cells and KIR/KIR ligand affinities (**I**) could be affected by both KIR and HLA class I polymorphisms.

Overall, the variability in KIR gene content, KIR allele polymorphisms, the HLA class I environment, and the clonal expression of KIRs result in the inter- and intraindividual diversity of the KIR^+^ NK cell repertoire (Figure 2).

## 3. Impacts of the KIR and HLA Class I Genes on the Antileukemic Effects of NK Cells in Patients with Acute Leukemia

### 3.1. Expression of HLA Class I Molecules on Leukemic Cells

Impaired recognition of tumor cells is associated with the loss of or decreased HLA expression [15]. This mechanism, which has been described in various solid tumors, could be related to genomic alterations of HLA genes, phenotypic alterations of HLA molecules, or changes in other elements involved in the antigenic presentation machinery. Toffalori et al. elegantly reported an immunological signature of relapse associated with a loss of HLA expression in AML patients [113]. In leukemic patients, the decreased expression of HLA class I molecules may depend on the loci, alleles, tissues, or cells, and it impacts different HLA molecules, leading to a loss of HLA heterozygosity or a complete HLA haplotype loss [114,115,116]. The nature of HLA class I downregulation also varies depending on the origin of the leukemia [117]. The decrease in HLA expression occurs most frequently at the time of relapse, thus constituting a potential immunological signature [118]. In 2009, after haplo-identical HLA HSCT, Vago et al. reported a loss of the mismatched HLA haplotype in one-third of the patients (mostly with AML) who relapsed [119]. In a collaborative study, at the time of diagnosis of AML patients, we reported a loss of heterozygosity in the complete HLA region in half of the patients, as well as the loss of the HLA-A, -B, and -C genes—or only the HLA-A gene—in the remaining patients [120].

The impact of the HLA-Cw expression level on clinical outcomes after mismatched HLA-C HSCT remains relatively undocumented. HLA-C mismatches are usually reported to be associated with a higher incidence of acute graft-versus-host disease (GvHD) and higher mortality [121]. However, some frequently encountered HLA-C mismatches, such as HLA-C*03:03/*03:04 or HLA-C*07:01/*07:02, which correspond to a low HLA-C expression level in recipients and are evaluated using the expected MFI of the corresponding HLA-C specificities, are called permissive in the absence of deleterious effects after unrelated mismatched HLA-C HSCT [122,123,124]. In contrast, no effects caused by HLA-C level expression, which was evaluated by using the polymorphism of a miRNA in the 3′UTR, were reported after an unrelated HSCT [125]. These discordances could rely on HSCT cohorts or methods to correlate the levels of HLA-C expression according to the HLA-C polymorphism. The level HLA-Cw expression on leukemic cells or on reconstituting immune cells after HSCT is poorly documented due to the lack of specific mAbs available. One could expect that the level of HLA-Cw expression could modulate NK cell functions and their development through their *trans*- and *cis*-interactions with KIRs, respectively.

### 3.2. Antileukemic NK Cell Function in Patients with Acute Leukemia

In the context of acute leukemia, NK-cell-mediated immunosurveillance requires the establishment of an immunological synapse between NK cells and the target; this synapse involves the interactions between inhibitory and activating NK receptors and their ligands expressed on leukemic cells. NK cells express a wide range of activating receptors that recognize induced or upregulated molecules on tumor cells [19]. Moreover, cognate ligands are heterogeneously expressed on leukemic cells. In addition to the absence of expression or downregulation of HLA molecules, it was reported that the presence of activating ligands on the surface of leukemic cells was associated with the reduction of relapse incidence and improved overall survival of patients with acute leukemia [126]. We recently reported that ALL targets were better recognized by NK cells than AML targets. However, there is a broad interindividual disparity in NK cell responses against the same leukemic target, which highlights bad and good NK responders. Most effective NK cell subsets against different ALL targets express NKG2A, and these represent the most frequent subset in the NK cell repertoire. In contrast, minority CD57^+^ and/or KIR^+^ NK cell subsets are more efficient against AML targets [127]. Overall, our data may help to optimize the selection of HSC donors on the basis of immunogenetic KIR/HLA markers and to identify the best NK cell candidates in immunotherapies for AML patients.

## 4. Selection of HSC Donors Based on the HLA and KIR Genes

### 4.1. Selection of HSC Donors Based on HLA Matching

**Figure 3:**
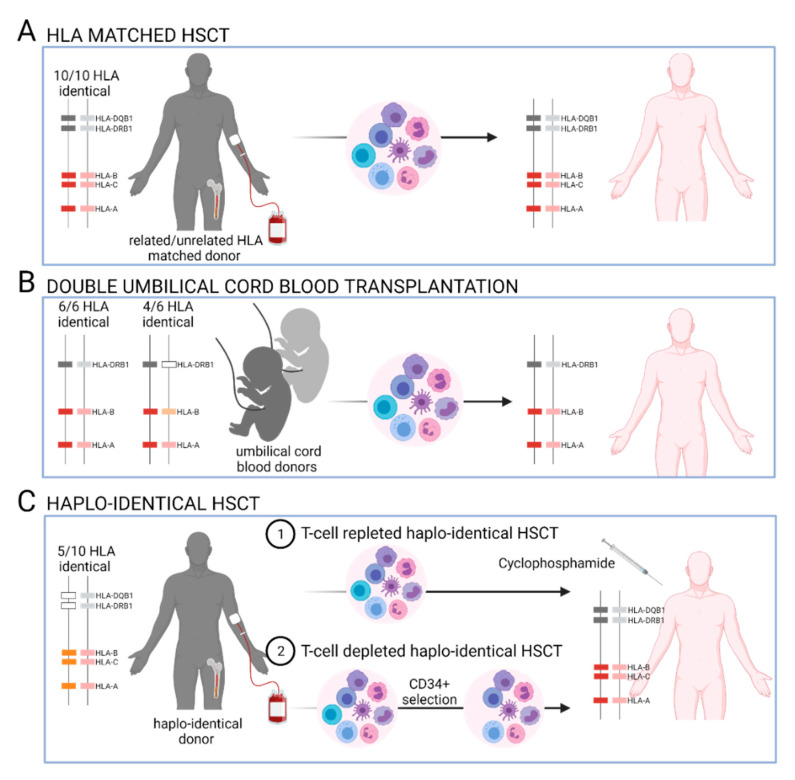
Donor/recipient HLA matching prior to allogeneic hematopoietic stem-cell transplantations (HSCT). The matching of the HLA-A, -B, -C, -DRB1, and -DQB1 genes between donors and recipients in both haplotypes is usually sought prior to related or unrelated HSCT (**A**). In the absence of related/unrelated 10/10 HLA-identical donors, double-umbilical cord blood transplantation (dUCBT) could be proposed so that only the matching of the HLA-A, -B, and -DR genes would be considered. In this context, the two UCB units and the recipient usually have 4/6 to 6/6 HLA matches (**B**). More recently, a donor with a 5/10 HLA-haplo-identical match is sought. The use of cyclophosphamide post-grafting as a prophylaxis against GvHD permits the deletion of the reconstitution of alloreactive T cells from T-repleted grafts (**C**).

The matching of HLA class I (HLA-A, -B,-C) and class II (HLA-DRB1, -DQB1) genes between the donor and the recipient remains the gold standard [128,129] in order to prevent acute GvHD occurrence, to improve overall survival (OS), and to provide a beneficial GvL effect that is driven by alloreactive T and/or NK cells. The inheritance of HLA genes from the parental haplotypes in blocks results in 25% likelihood of finding a 10/10 HLA-identical donor among a patient’s siblings. In the absence of related HLA-identical donors, an unrelated HLA-matched donor is sought in the registries. In an unrelated HSCT, an important feature to consider is that the HLA alleles, haplotype frequencies, and LD between HLA genes may differ across populations [63]. Although there are more than 30 million potential unrelated HSC donors currently registered worldwide, finding suitably matched donors can be difficult for patients that harbor rare HLA alleles and/or because of the quality of available genotyping information. In the absence of a related or unrelated 10/10 HLA-matched donor, a 9/10 HLA-matched donor with selective mismatches could be selected. From 2010 to 2015, umbilical cord blood donors represented another option. For adult patients, two UCB donors were selected, and the level of HLA matching between the donor and recipient in double umbilical cord blood transplantation (dUCBT) was based on the HLA-A, -B, and -DRB1 genes (4/6 to 6/6 HLA matched). The impact of HLA-C matching remains to be clarified. Over the last 5 years, unmanipulated T-cell-repleted haplo-identical HSCT using cyclosphosphamide post-transplantation (haplo-PTCy) as prophylaxis against GvHD has been recommended in the absence of a 10/10 HLA-identical donor. Haplo-PTCy is increasing in use and has shown similar outcomes to those observed after unrelated or even related HLA-matched HSCT [130,131]. Here, donors and recipients share only one HLA haplotype, resulting in a 50% likelihood of finding a haplo-identical donor among one’s siblings and, therefore, increasing the number of potential HSC donors. The levels of HLA matching between a HSC donor and a recipient depending on the stem-cell sources are summarized in Figure 3.

### 4.2. KIR Models Used to Evaluate NK Cell Alloreactivity and Clinical Outcomes after Allogeneic HSCT

**Figure 4:**
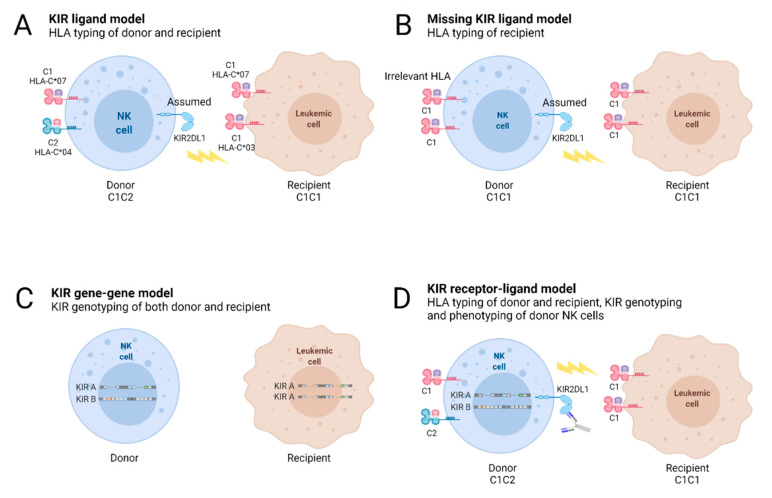
Genetic KIR/KIR ligand models defined to predict potential KIR^+^ NK cell alloreactivity (adapted from Symons et al. [134]). Genotyping of HLA class I and/or KIR genes permits the establishment of different KIR/KIR ligand models for use in HSCT. Knowledge of the HLA class I typing of donor/recipient pairs permits the identification of KIR ligand mismatches in the graft-versus-host (GvH) direction in HLA-mismatched HSCT, as defined by the KIR ligand model (**A**) or by the KIR ligands in recipients that show missing KIR ligands (**B**). In these contexts, the expression of KIR is only predictive. KIR genotyping of donors and recipients permits the assignment of donor and/or recipient KIR genes that impact the HSCT outcome, as defined by the KIR gene/gene model (**C**). Genotyping of HLA class I genes in donor/recipient pairs and of KIR genes in donors permits the identification of both educated KIR^+^ NK cells and missing KIR ligands in recipients (**D**). In this KIR receptor–ligand model, the expression of KIRs could be assessed using appropriated anti-KIR monoclonal antibodies (mAbs) or could be deduced from the donor’s KIR alleles (**D**).

To evaluate the potential alloreactivity of KIR^+^ NK cells, the impacts of KIR ligands, KIR genes/genotypes, and KIR/KIR ligand (KIR-L) mismatches have been deeply investigated since 2000 in terms of HSCT outcomes; due to the heterogeneity of the HSCT cohorts and/or the predictive KIR models used, the results were discordant (Figure 4) [132,133].

The beneficial effect of NK cell alloreactivity based on HLA class I mismatches in terms of KIR ligands in the graft-versus-host direction (GvH) was initially underlined in the context of T-cell-depleted haplo-identical HSCTs, specifically for AML patients without relapse and aGvHD [135,136]. In parallel, the impacts of KIR ligands, donor KIR genes, and KIR-L mismatches on clinical outcomes were investigated after related or unrelated HSCTs, including HLA-matched and HLA-mismatched pairs. Notably, as HLA class I and KIR genes are located on different chromosomes, HLA-matched donor/recipient pairs could present relevant KIR gene mismatches. In related HLA-matched HSCT, the absence of KIR ligands in the recipient (missing KIR ligand, i.e., the absence of Bw4, C1 or C2) for the donor’s inhibitory KIRs was found to either be a beneficial prognostic factor by increasing OS and disease-free survival (DFS) and/or by decreasing relapse incidence for AML patients [137,138] or have no effect [101]. In addition to genetic studies of KIR, different profiles of NK cell reconstitution were reported with the establishment of a donor-like KIR repertoire, showing a gradual reconstitution from 6 to 9 months or longer to 1 year after related HLA-matched HSCT [137]. NKG2A^+^ KIR^−^ NK cells were shown to dominate early after HSCT, and NK cells that lacked inhibitory KIRs for self-HLA class I ligands remained tolerant at different points in T-cell-repleted and -depleted HLA-matched sibling HSCTs [101].

In unrelated HSCT, having a missing KIR ligand was found to be a predictive factor for relapse in adult patients with AML in HLA-mismatched pairs [139]. Interestingly, in a large series of unrelated HSCTs, including HLA-matched and single-mismatched pairs, Venstrom et al. reported a reduction in relapses in AML patients that were grafted with KIR2DS1+/C1+ donors [140]. In addition to KIR2DS1, we reported a beneficial impact of KIR3DL1/S1+ grafts in Bw4+ recipients with myeloid diseases on relapse incidence after unrelated HSCT [141]. Venstrom et al. also confirmed the beneficial impact of KIR3DS1+ donors by decreasing treatment-related mortality (TRM) and aGvHD, but found that there was no impact on relapses [142]. More broadly, a specific donor KIR B gene content score that is ≥2 [143], donor KIR B genotypes [144], or donor KIR CenB02 [145] should be favored for AML patients in order to decrease relapse incidence, which suggests that there is a need to detect the activating KIR genes. Notably, one prospective and multicentric study underlined the feasibility of donor KIR genotyping without delaying grafting [146]. In contrast, the assignment of donor KIR alleles is more time-consuming, and their effect on HSCT outcomes is primarily focused on limited KIR genes with phenotypic and functional characteristics of KIR^+^ NK cells, as was reported for KIR2DL1 [147]. Interestingly, a beneficial effect of specific donor KIR3DL1-HLA-B subtypes associated with weak or a lack of inhibition of NK cells was reported in a large series of AML patients that included unrelated 10/10 and 9/10 HLA-matched pairs [148]. Conversely, the genetic combination of donor KIR3DL1+ and patient HLA-A*24+ is associated with a higher risk of AML relapse following allogeneic HSCT [149]. Schetelig et al. reported that there were no correlations between KIR3DL1/HLA-B combinations and relapse or OS in unrelated HSCTs, which also included 10/10 and 9/10 HLA-matched pairs [150]. Recently, a retrospective study performed on a large cohort of AML patients reported that there was no impact of KIR3DL1/2DS1 or a haplotype-motif-based donor selection algorithm on OS and relapse after unrelated HLA matched HSCTs [151]. In line with HSCT protocols, the impact of KIR-L mismatches was investigated in adult patients after dUCBTs [152,153,154,155,156,157,158]. Once again, conflicting data were reported, which were linked either to the competing KIR models used or the heterogeneity of the patients and treatment modalities.

For the last 5 years, haplo-PTCy has offered a new means of identifying the KIR and/or HLA class I markers that are beneficial in improving the GvL effect driven by NK cells [159,160]. In this context, donors and recipients share only one HLA haplotype; thus, HLA class I mismatches could represent KIR-L mismatches. Donors and recipients could also have different KIR genes. In terms of inhibitory KIR gene mismatches, Symons et al. [134] reported a beneficial effect on the improvement of OS and the decrease in relapse and non-relapse mortality (NRM) after haplo-PTCy in 86 patients with lymphoid and myeloid diseases. Bastos-Oreiro et al. [161] reported the beneficial effect of at least one inhibitory KIR gene mismatch on the improvement of event-free survival (EFS) and the decrease in relapses after haplo-PTCy in 33 patients with various diseases. However, the specificity of the inhibitory KIR gene mismatches was not mentioned. Wanquet et al. [162] reported decreased relapses in patients with active diseases with KIR-L mismatches, but there were no beneficial effects of these mismatches for patients in complete remission after haplo-PTCy. Solomon et al. [163] reported a beneficial effect of KIR-L mismatches on OS and relapses after haplo-PTCy in 208 patients that received either a myeloablative conditioning (MAC) or a reduced-intensity conditioning (RIC) regimen. In 51 patients, which were all treated with an RIC regimen and infused with PBSCs, we reported a beneficial effect of KIR2D/HLA-C mismatches in the GvH direction, by decreasing the relapse incidence while increasing OS, aGvHD, and DFS after haplo-PTCy [164]. In contrast, in AML patients grafted with PBSCs, Shimoni et al. [165] reported a deleterious effect of KIR-L mismatches with increased relapses and decreased OS, after haplo-PTCy. In terms of KIR genotypes, Symons et al. [134] showed in 86 patients that KIR genotype AA recipients from B^+^ KIR donors were associated with fewer relapses. The beneficial effect of donor B^+^ KIR was reported in both lymphoid and myeloid diseases and with RIC regimens [134]. Solomon et al. [163] reported a beneficial effect of the donor B^+^ KIR genotype on relapse incidence, particularly the presence of the KIR2DS2 gene in 208 patients with various diseases. Mixed bone marrow/PBSCs and both MAC and RIC regimens were used in these series. Lastly, Bastos-Oreiro et al. [161] reported that there was no protective effect of the donor B^+^ KIR genotype on relapse incidence after haplo-PTCy in a limited number of patients with mixed conditioning regimens. In terms of KIR gene motifs, we recently reported the impact of the donor KIR cenAA motif on relapse incidence after haplo-PTCy in 39 patients with myeloid diseases, which suggested that the GvL effect could be driven by specific KIR2DL1/3 allotypes [34]. The impact of KIR allele polymorphisms on haplo-PTCy outcomes has not been investigated so far.

Overall, these studies, which were mainly retrospective, demonstrated that the impacts of genetic combinations of KIR ligands, KIR genes, and/or KIR-L differ depending on diseases, immunosuppressive treatments, conditioning regimens, the stem-cell sources, the graft T-cell content, the GvHD prophylaxis, and the degree of HLA matching. However, HLA typing alone is not sufficient for providing the specificities of the donor KIR genes involved. Although the gene frequencies of KIR2DL1/3/3DL1 are close to 100%, allowing speculation about their presence depending on donor KIR ligands, the distinguishing between KIR2DL2 and 2DL3 is essential because their phenotypes and functions in NK cells differ [81]. KIR3DL1 allele typing also permits one to check for the absence of 3DL1*004, which is frequent in Caucasian people [46]. The reported and divergent effects of KIR/KIR ligand genetic combinations on HSCT outcomes (Table 1) suggest that donor KIR genotyping, including the detection of activating KIR genes, discrimination among KIR gene motifs, KIR allele polymorphisms, and the HLA class I environment, should be considered in order to predict the potential KIR^+^ NK cell alloreactivity after HSCT. Given the current literature, it is proposed that an HSC donor with B+ KIR gene content is selected for HLA-identical HSCTs, and an HSC donor with a KIR cenAA motif is selected for haplo-PTCy in order to limit the relapse incidence in adult patients with AML.

### 4.3. Reconstitution of NK Cells after T-Replete Haploidentical HSCT

After haplo-PTCy, NK cells rapidly reconstitute the recipient’s immune system, as they are the first lymphocytes to mediate a GvL effect, even before T cells [164,166,167,168]. The prophylaxis against GvHD plays a major role in the modulation of the reconstitution of immune cells and possibly in their function after haplo-HSCT. Recovery of NK cells after haplo-PTCy was reported by Vago’s group in 2018 [168]. A similar NK cell recovery was observed in patients who received different PTCy protocols. PTCy first eliminates proliferating donor NK cells; then, an IL15 peak correlated with the kinetics of NK cell recovery appears, and a second wave of reconstituted NK cells with an immature phenotype occurs 2 weeks after HSCT. The development of mature NK cells can take place up to 1 year after HSCT. PTCy decreases NK cell alloreactivity, and the GvL effect is correlated with residual mature NK cells. Overall, there are predictive factors that correspond to the absolute counts of NK cells and the proportion of mature NK cells at day +30. A high expression of KIRs on NK cells at day +30 was correlated with a decreased relapse incidence [168]. Consistently with the data from Russo et al., we evaluated NK cell reconstitution in a cohort of 51 adult patients after haplo-PTCy [164]. KIR2DL2/3^+^ and 3DL1^+^ NK cell recovery at day +30 was inversely impacted by KIR-L mismatches. The loss of KIR2DL2/3^+^ NK cells at day 30 in pairs with inhibitory KIR2D/HLA-C mismatches suggested a deletion of alloreactive KIR^+^ NK cells by PTCy [164]. After haplo-PTCy, the reconstitution of T and NK cells revealed increased NK and decreased T-cell frequencies in patients treated with PTCy + ATG compared to PTCy alone [167]. In patients without relapse, the frequency of NKG2A^+^ KIR2DL2/3/S2^−^ NK cells was increased at day +60 and these cells were more numerous in patients with PTCy + ATG compared to PTCy alone. The use of the NKG2A, CD57, NKG2C, and KIR2DL3 markers expressed on NK cells at day +30 made it possible to define an immature NK cell repertoire (i.e., KIR2DL3^−^NKG2A^+^CD57^−^) associated with fewer relapses [167]. In contrast, the predominant KIR2DL3^−^NKG2A^−^CD57^−^ NK cell subset was associated with a high incidence of relapse [167]. Overall, deciphering the heterogeneity of NK cells is crucial for identifying the NK cell subsets that are efficient against hematological diseases in order to optimize HSC donor selection and, more broadly, NK-cell-based immunotherapies, as discussed below.

## 5. NK-Cell-Based Immunotherapies Apart from HSCT

In leukemic patients, the functions of NK cells are usually impaired, which is particularly linked to the alteration of their repertoire. Thus, NK-cell-based immunotherapies are devoted to increasing their antileukemic potential in patients by using the addition of specific cytokines, mAbs, and/or the adoptive transfer of unmodified or engineered NK cells [18,19,169,170,171,172,173,174].

### 5.1. Immune Molecules for Boosting NK Cell Functions in Leukemic Patients

Among the various cytokines, IL-2 and IL-15 are of interest for promoting the survival, proliferation, differentiation, and activation of NK cells. In particular, ALT-803, a super-agonist of IL-15 that was administrated to 33 patients who relapsed after HSCT, showed a clinical benefit in 19 patients in a phase I trial [175]. In clinical studies that combined ALT-803 with adoptive transfer of NK cells, ALT-803 was expected to enhance the activation, survival, and expansion of donor-derived NK cells. Moreover, the cocktail of IL-12, IL-15, and IL-18 used in vitro to promote NK cell proliferation and activation unleashes tumor killing of NK cells via KIR downregulation [176]. Apart from cytokines, mAbs that are specific for tumor antigens could be used to enhance the ADCC function of NK cells through the binding of the IgG Fc fragment with the CD16 receptor. In particular, an Fc-engineered CD133 mAb showed an improved affinity to NK cells and potent NK cell degranulation against AML [177]. Among mAbs, bi- or tri-specific killer engagers (BiKEs, TriKEs) are used to modulate NK cell activity. They are designed to contain variable single-chain fragments that function against both tumor-associated antigens and NK-activating receptors in order to create an immunological synapse between NK cells and tumor cells [178]. In particular, it has been reported that the CD16x33 BiKE targets the CD33 myeloid antigen and triggers the lytic activity of NK cells and the release of cytotoxic granules against AML cells [179] in vitro. Moreover, the 161533 TriKE, which consists of an anti-CD16 scFv, a modified IL-15 linker, and an anti-CD33 scFv, could trigger ADCC while increasing both NK cell survival and proliferation against AML targets in vivo [180]. NK-cell-based immunotherapies using mAbs could also target immune checkpoints to raise the inhibition driven by NK cells. Interestingly, lirulimab and monalizumab target the pan-KIR and NKG2A inhibitory NK cell receptors, respectively [181]. PD-1 could also represent an interesting target [171]. KIR/HLA interactions could also have an impact on the ADCC function of NK cells, as we recently reported in lymphoma patients who received a rituximab-based anticancer immunotherapy [182].

### 5.2. Adoptive NK Cell Immunotherapies

**Figure 5:**
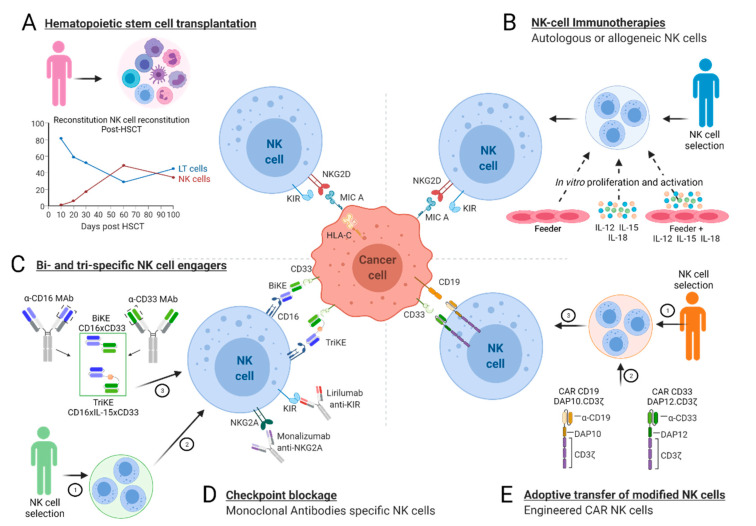
NK-cell-based immunotherapies for acute leukemia patients. Because of their antileukemic (GvL) effects, NK cells play a major role, as they are the first cells to reconstitute the recipient’s immune system after allogeneic hematopoietic stem-cell transplantation (HSCT) (**A**). Apart from HSCT, the efficiency of NK-cell based immunotherapies could be enhanced with the infusion of autologous or allogeneic NK cells that are activated in vitro using a feeder and/or specific cytokines (**B**). The use of bi- or tri-specific killer engagers (BiKEs, TriKEs) (**C**) or specific anti-NK cell receptor monoclonal antibodies (mAbs) (**D**) could also enhance the GvL function of NK cells. Adoptive transfer of engineered NK cells armed with chimeric antigen receptors (CARs) (**E**) is another emerging alternative.

Infusions of allogeneic NK cells that are activated ex vivo or CAR-modified NK cells represent promising immunotherapies against hematologic malignancies. In contrast to T lymphocytes, allogeneic NK cells are not HLA-restricted and can be more safely used. Another advantage is the wide range of NK cell sources such as PB, UCB, embryonic stem cells, induced pluripotent stem cells, and NK cell lines [183,184]. In several clinical trials of NK-cell-based immunotherapies, patients received chemotherapy prior to NK cell infusion. Interestingly, the drugs used could stimulate the expression of activating ligands or either downregulate the expression of inhibitory ligands on tumor cell [185] or promote the recruitment of NK cells to the tumor sites by increasing the secretion of selected chemokines. In addition to the adoptive transfer of unmodified NK cells, CAR NK cells could be used. Similarly to CAR T cells, CAR NK cells are composed of an extracellular signaling domain that recognizes specific antigens on tumors, a transmembrane region, and an intracellular domain that activates pathways to enhance the lysis of target cells. CAR NK cells eliminate tumors not only through the ability of CAR to specifically recognize antigen-expressing tumors, but also through NK cell receptors themselves [20]. Preclinical trials of NK cells demonstrated their clinical benefits in AML patients after HSCT. However, the clinical activity of NK cells remains modest at best, which limits their current therapeutic use. CAR-modified NK92 and primary NK cells are currently used against hematological cancers. The tumor antigen that is targeted is often CD19 or CD20, which makes it possible to target B-cell malignancies, such as B-ALL or CD33, in order to target AML [19]. However, some issues related to the survival rate and cytotoxicity of NK cells after thawing need to be solved in order to expand the use of CAR NK cells in clinical applications. New strategies, including the integration of suicide genes into CAR constructs and bispecific CAR molecules, are being developed to increase both the safety and the efficacy of CAR NK cell therapy [20]. The NK-cell-based immunotherapies used for the treatment of acute leukemia are summarized in Figure 5.

Overall, NK-cell-based immunotherapies could be combined to boost the efficacy of NK cells against leukemia, depending on the patients. However, given the heterogeneity of NK cells, the source of NK cells is still a key element, and allogeneic donors should be carefully selected by using specific KIR/HLA markers.

## 6. Conclusions

Polymorphic KIR and HLA class I genes play a major role in the structuration of the functional NK cell repertoire. In the context of HSCT, they are of special interest for the promotion of a beneficial GvL effect in leukemic patients. Haplo-PTCy makes it possible to choose the best HSC donor among different ones. Until now, few studies have focused on the relevance of polymorphic KIR/KIR ligands to the outcomes of haplo-PTCy. One could expect that the consideration of the KIR gene content or specific KIR alleles could help to refine the scores used for HSC donor selection, as well as to evaluate their influence on haplo-PTCy outcomes in patients with high-risk and acute leukemia. The ultimate goal is to define the best KIR and/or HLA markers during HSC donor selection with respect to a beneficial GvL effect and a reduced GvHD incidence after haplo-PTCy. More broadly, the inclusion of KIR/HLA markers should improve the efficiency of allogeneic NK-cell-based immunotherapies, such as treatment with CAR NK cells.

## Figures and Tables

**Table 1:** Impacts of the genetic combinations of KIR/KIR ligands on clinical outcomes for adult AML patients, including selected studies that focused on both HLA-matched and HLA-mismatched HSCTs.

Authors	*N*	AML ^1^	HSCT	HLA	Source ^2^	Depletion	Conditioning ^3^	Outcome ^4^
Hsu et al. [138]	178	32%	Related	Matched	BM	T-cell-depleted	MAC	AML patients with missing KIR ligands (HLA-C or HLA-B ligands): ↗ OS, ↗ DFS, ↘ relapse
Björklund et al. [101]	105	77%	Related	Matched	PBSC (67%) and BM (33%)	T-replete	MAC (72%) and RIC (28%)	No benefit of missing KIR ligand on DFS, relapse, and aGvHD
Hsu et al. [139]	1770	13%	Unrelated	Matched (48%) and mismatched (52%)	unknown	T-replete	MAC	AML patients with missing KIR ligand: ↘ relapse in mismatched HSCT but no impact on matched HSCT
Venstrom et al. [140]	1277	100%	Unrelated	Matched (52%) and mismatched (48%)	BM (54%) and PBSC (46%)	T-replete (72%) and T-cell-depleted (28%)	MAC (85%) and RIC (15%)	Donor 2DS1+/C1+: ↘ TRM, ↘ relapse
Gagne et al. [141]	264	52% of acute leukemia	Unrelated	Matched (62%) and mismatched (38%)	BM	T-replete	TBI based (70%)	Donor KIR3DL1/S1+ and recipient Bw4+: ↗ OS, ↘ relapse for HLA-matched and HLA-mismatched HSCTs for malignant diseases
Venstrom et al. [142]	1087	28%	Unrelated	Matched (62%) and mismatched (38%)	BM (97%)	T-replete (80%) and T-cell-depleted (20%)	Unknown	Donor KIR3DS1+: ↘ TRM, ↘ aGvHD, no impact on relapse
Cooley et al. [143]	1409	77%	Unrelated	Matched (50%) and mismatched (50%)	BM (90%) and PBSC (10%)	T-replete	MAC	Donor CenBB or ≥ 2 KIR B gene content: ↗ DFS and ↘ relapse in matched and mismatched HSCTs.Donor TelBB: ↘ relapse
Weisdorf et al. [144]	2662	100%	Unrelated	Matched (89%)	BM (15%) and PBSC (85%)	Unknown	MAC (59%) and RIC (41%)	Donor KIR B haplotype: ↘ relapse only in RIC patients. Donor KIR B genes/ recipient C1+: ↘ relapse
Boudreau et al. [148]	1328	100%	Unrelated	Matched (53%) and mismatched (47%)	BM (54%) and PBSC (46%)	T-replete (92%)	MAC (84%) and RIC (16%)	Donor KIR3DL1/HLA-B subtype combinations linked to weak or non-inhibition: ↘ TRM, ↘ relapse. Distinct beneficial effect of donor KIR2DS1+/C1+
Ploeg et al. [149]	604	100%	Unrelated	Matched (64%) and mismatched (36%)	BM (48%) and PBSC (52%)	T-replete (67%) and T-cell-depleted (33%)	MAC (85%) and RIC (15%)	Donor KIR3DL1+ and recipient HLA-A*24+: ↗ relapse
Ruggeri et al. [135]	92	62%	Related	Haplo-identical	unknown	T-cell depleted	Unknown	Donor/recipient KIR ligand mismatches in the GvH direction: no aGvHD and no relapse in AML patients
Symons et al. [134]	86	30%	Related	Haplo-identical	BM	T-replete	RIC, PTCy	Donor/recipient inh.KIR gene MM: ↗ OS, ↘ relapse, donor KIR B/recipient KIR AA ↗ OS, ↘ NRM
Bastos et al. [161]	33	30%	Related	Haplo-identical	unknown	T-replete	RIC (66%) and MAC (34%), PTCy	At least one donor/recipient inh KIR gene MM: ↗ EFS, ↘ relapse. No protective effect of donor B genotype
Wanquet et al. [162]	144	35% of myeloid diseases	Related	Haplo-identical	PBSC (63%) and BM (37%)	T-replete	RIC (87%), MAC (13%), PTCy	Donor/recipient KIR ligand MM in the GvH direction: ↘ relapse, ↗ PFS for patients with active disease but no beneficial effect for patients in complete remission
Solomon et al. [163]	208	34%	Related	Haplo-identical	PBSC and BM	T-replete	MAC (41%), RIC (59%), PTCy	Donor/recipient KIR ligand MM in the GvH direction or donor KIR2DS2+: ↗ OS, ↗ DFS, ↘ relapse. No impact on aGvHD
Shimoni et al. [165]	444	74%	Related	Haplo-identical	BM (53%) and PBSC (47%)	T-replete	MAC (55%) and RIC (45%), PTCy	Donor/recipient KIR ligand MM in the GvH direction: no impact on aGvHD, cGvHD and NRM. In AML patients grafted with PBSCs, ↗ relapse and ↘ OS
Willem et al. [164]	51	35%	Related	Haplo-identical	PBSC	T-replete	RIC, PTCy	Donor inh.KIR2D/recipient HLA-C MM: ↗ aGvHD, ↗ OS, ↗ DFS, ↘ relapse
Dubreuil et al. [34]	81	63% myeloid diseases	Related	Haplo-identical	PBSC	T-replete	RIC, PTCy	Donor CenAA: ↘ relapse only in patients with myeloid diseases

^1^ AML, acute myeloid leukemia; ^2^ BM, bone marrow; PBSCs, peripheral blood stem cells; ^3^ MAC, myeloablative conditioning; PTCy, post-transplant cyclophosphamide; RIC, reduced-intensity regimen; ^4^ aGvHD, acute graft-versus-host disease; CenAA, centromeric A KIR motif; CenBB, centromeric BB KIR motif; DFS, disease-free survival; HSCT, hematopoietic stem-cell transplantation; inh. KIR, inhibitory KIR; MM, mismatch; NRM, non-relapse mortality; OS, overall survival; PFS, progression-free survival; TRM, treatment-related mortality.
